# Odontological Society of Pennsylvania

**Published:** 1886-08

**Authors:** 


ARTICLE III.

### ODONTOLOGICAL SOCIETY OF PENNSYLVANIA

The regular meeting of the Odontological Society of Pennsylvania was held Saturday evening, November 7th,

1885, at the office of Dr. Tees, 548 North Seventeenth street, President Darby in the chair. The following paper was read:

### THE PHILOSOPHY OF THE TOOTH-BRUSH—ITS CONSTRUCTION AND PROPER USE.

BY W. G. A. BONWILL, D. D. S.

I am not aware that any one has ever written a dissertation upon this subject. There has been some talk on the hygiene of the mouth, during the past year, but no one has attempted to explain the philosophy of a tooth-brush: how it should be constructed, and its proper use. It is a lamentable fact that but little thought and ingenuity has ever been given to it, although thousands of shapes have been furnished by dentists and others. I must confess to my own shortcomings, up to the year 1876. I had not, up to that date, ever sold brushes, powders or washes from my office. It looked too much like trading. I always told my patients to go to drug stores and select for themselves.

My attention was arrested, in 1876, by two remarkable cases: Two bachelor brothers had left their dentist on account of not being able to arrest the disease which, he said, was fast cutting their teeth away, both upper and lower. It was noticed that a groove near the cervix on the buccal and labial sides, as far back as the second molars, was being cut. The dentist said it was a disease, and more care must be taken. The brush, with washes and powders, must be applied thrice daily. The apparently specific disease spread, in epidemic form, with both brothers. Filling was resorted to, but with little good results. It was looked upon as an incurable disease, the only remedy being brushing.

When they came to me they were frantic.

In making my diagnosis, I was satisfied, from my previous experience, that the whole trouble arose from the injudicious use of the brush and powders. They spent fifteen minutes after each meal in this work of preservation, as they thought, and once before retiring. They seemed to have nothing to do but think of their teeth and how they should avert the calamity impending. The filling that had been done was well consolidated, and calculated to have saved from action of caries alone. But nothing could withstand such friction as had been kept up for years. The bristles did not cut the gold, but the enamel went on to destruction around the edge of the fillings. The younger brother, about forty-five years of age, had lost the six superior incisors from having been cut so far through from the labial side that they broke off; and the lower were gone so nearly to the pulp canal as to endanger them. In fact, wherever he could bring the brush into action by abrasion across the surface of all his teeth, even upon the palatal and lingual sides, the effect was evident.

I had seen many cases where the teeth had been abraded, but these surpassed all.

In making up my decision, it was necessary to determine whether there could be such a factor as disease, in such cases, or whether it was from the brush and dentifrices, with washes, &c. Some of the best practitioners had held that there was some specific action going on in the secretions of the mouth, *but had never given the crucial test to substantiate their assertions.* My own experience. hitherto, had fully assured me that there was but one cause—the *Brush* and the incidentals. I could see nothing else, in these cases, and so decided. I at once changed the whole state of affairs. Of the results of my diagnosis and prognosis I had no doubt. I selected them a small brush, and, as a powder, prepared chalk. They were directed to use the brush by simply placing it in one spot and pressing the bristles directly between the teeth with a rotation of the brush; not to rotate the wrist and brush up and down from the gums to cutting edges; avoiding the old method of drawing the brush across the teeth; not to consume more than two minutes after breakfast and supper, using powder but once daily. The denuded of abraded teeth were contoured with gold (Abbey's No. 5, soft,) and by hand pressure. I have seen them twice yearly, ever since 1876, and I can assure you that no recurrence or spread of the trouble has taken place in the least.

There were many such cases about that time coming to me, and I felt it a duty to construct a brush which, from the study of these phenomena, would obviate or anticipate it. While I differ with many of you, I must repeat that I *have never seen a case where the teeth had been generally eroded, but could be accounted for by friction, and nothing else.*

There is one particular form of groove that has caused so many to believe it was from disease. It seems easy to attribute it to the brush, so long as the groove is a shallow concave; but where the edge of the groove, on the side nearest the cutting edge, is an acute angle, and forming a sharp corner, the brush is not a factor. It would seem impossible to make such a shaped groove with a yielding substance like bristles. When it is carefully studied, however, one must admit it possible and certain. The brush, in nearly every case, has been driven across the surfaces of the teeth, in conjunction with powder, with a great force exerted upon the bristles, to press them upon the teeth, and produce friction. A groove, it will be admitted, is soonest made near and at the cervix by first wearing away the gum, when the bristles can touch the dentine. In many cases of rather flat teeth, the groove is lower. Sometimes not a groove, but indentation on the surface, with intervening patches of hard enamel untouched, or only partially so.

Now, when you consider how much force has to be brought to bear to keep the brush in contact with the teeth, and the brush being too wide and too long, with the bristles too close together, if a groove is made at all, then, as soon as commenced, and an edge or boundary line is made, the bristles can no longer keep on the line, but divide, and the body of the bristle—not its end or point—is constantly in friction against this line, making it at first as sharp as a razor, and keeping it so. If you could keep the bristles on this brink of the groove, then it could not occur.

Another reason why the groove commences at or near the cervix is, that there is less resistance to the brush from the neck being narrower; besides, there is a less body of the tooth presented. It is there the gum forms a boundary line to control the bristles, to keep them in file in their ranks, standing up squarely to their work, while those on the flatter body of the tooth go straddling around as if intoxicated. Then, as soon as the slightest groove is made, the bristles are bound like men to go in the *rut*. When the cuspids, bicuspids, and molars are the prey, you never see these grooves except close under the edge of gum, never upon their broadest surfaces. There is one phenomenon that seems not to be in keeping with this explanation and it is the unfathomable paradox to all who hold to a specific cause. How is it possible while the brush is made to pass across the surfaces of the incisors that, in addition to a groove or grooves, there should be a hill and plane surface produced? Why not the groove, as near the cervix. This is not at all wonderful. I am quite sure that no round face tooth was ever so defaced. That it is to be found only in those cases where the enamel has given previous evidence of its surface being made up of cells of unequal value, which we so often witness? Who has not observed the surfaces of the hardest rock subjected to the constant attrition of water, and the debris therein, cut in a similar manner, whether the grain of the rock was lying lengthwise or crosswise. Something must give way where a force is exerted; and here, where the face of the tooth is flat and the bristles can be kept upon the surface without straddling, if one portion is softer than another there will be loss of structure irregularly. These reasons are sufficient to convince me that no other cause need be given for these phenomena. As soon as the excavated surfaces are filled and the brush used in moderation and with judgment, there will be no recurrence in any case.

You will understand from what has been said that I consider all cases where the labial, buccal and palatial surfaces are denuded, either in grooves, or when the broad surfaces of the centrals are involved near the cutting edge, it must be attributed to the brush and powders; yes, to washes also indirectly. You will never find it where a brush has not been used. The only thing answering to the specific theory is around the cervix where the brush has first worn the gum away, and caries taken the place of the brush, which latter can no longer be used without pain, and the parts are neglected. But, it is of an entirely different nature from the hard polished surfaces from attrition. While these surfaces are not so polished as those lower down toward the cutting edges, yet, they are partially so and the dentine is harder, nevertheless, it can be arrested at once by proper filling, as can all evaded surfaces, if the brush is also properly used. It matters not whether from disease or by friction, the remedy is all the same and equally effective. If it were caused by specific action nothing mechanically would avail. If I am not correct in my view and a specific cause is at work, will it make the case any better by continuing to use the brush either across or up and down? Not at all. Take for granted we know no cause for this vandalism. Can we not institute some more philosophical means of grappling with this monster? Is there a brush that has ever been in the market or in the private practice of any dentist which has any claims to a construction that will reasonably do the work for which they were designed and not cause mischief? I unhesitatingly answer, No!

What are the issues to be met? Will any one here say that, with the brushes to be had he can cleanse the teeth without producing *pressure*, and, the resultant *friction*. All will admit that, if the teeth can be cleansed, and well up under the cervical edges, without any possibility of abrasion on the enamel and lacerating or wearing away the gum, it should be the practice. Can we hope to do this with such brushes as we are compelled to buy? It is absolutely impossible, without the greatest care and vigilance, to guard against the crosswise movement which is first learned in youth. It is kept up through thoughtlessness, the dentist, until within a few years, seldom called attention to the practice. It becomes a second nature and, when they *are* advised as to the extent of the mischief done by their suicidal hands, it is frequently too late.

Then to cleanse the teeth, *we must produce more or less friction*, how shall it be done with the least pressure upon the handle of the brush, and what style or construction shall it have? And further, shall any powder or wash be used, and if so in what form? Should a wash ever be sold by any dentist or advised? Let us look at the construction of the average brush. The handle is evidently made to allow the patient to grasp it with the whole hand; for without this, it could not be held steady with such a mass of bristles at the opposite end. Look at the arrangement of those bristles. Four and five rows in width and seldom less than two inches in length and every bundle of them in holes as close as soldiers in solid column, ranging from soft to very hard, and no allowance in quantity made for the difference in their flexibility. Did you ever for a moment stop to think that such a mass was never designed to cleanse the human teeth or those of any of the lower animals? Reflect how much pressure must be exerted on such a solid phalanx to drive them between the teeth! Should you be able to get them, there, at what expense must it be upon the gums from the direct pressure alone; and, if any movement is made to create friction, the pressure must be con stantly kept up or the bristles will not, for a moment, remain, except upon the periphery of the tooth opposite the radius. You see now how in keeping the large handle is with the necessary force to be exerted from the dense column of bristles forming the appartus designed for the end in view. How few of the bristles can be expected to reach their destination by any movement possible? The friction of course is immense. How much of the length of the standard brush at any one time, or at all ever comes in contact with the teeth? In a word, how much material do you suppose can be dispensed with in making of a brush and be more effective? How much smaller and shorter can the handle be and give you control over the bristles while in the mouth? How much less friction can be produced for all practical purposes without so much pressure as hitherto exerted upon the standard brush? How much time can be saved? How much more pleasure can be assured? How much greater inducement, can be offered to those who now consider it a nuisance to brush their teeth? How much less risk can be guaranteed to those who know they have rather invited caries by their life-long attention than they have benefitted? How are we to please the baby and the older child and not disgust them with such an enormous mouth full of what is more fitted for scrubbing steps than human teeth. Had I not fancied I could answer all these queries in the affirmative I should not have troubled myself to tax your weary brains with the result of six years labor and observation; I cannot let the matter pass without doing what I consider a duty and a privilege.

Then, if *friction* must be produced in order to cleanse the teeth shall it be applied with a large or small brush; one thickly studded with bristles or one with them far apart in length and width? Shall it be applied crosswise or lengthwise the axis of the tooth? Shall we use powder or washes and how often and what kind?

As the teeth are of various shape and all of them with rounded faces. I claim it is impossible to use the standard brush, even once a day without injury being done to both teeth and gums. In the first place then, as we must reach all the surfaces of the teeth, I adopt a stiff bristle and set it in rows lengthwise, double the distance apart ot any other brush, and only long enough to cover about four teeth. There are three rows crosswise, and the holes in the handles are drilled to make the bristles converge or lean toward the centre, occupying but a trifling width on the face of the brush. Each row of bristles is pointed to give least resistance. The face of bristles, are again rounded from side to side to prevent them from touching the gums, before the teeth. The handle is made short and small in every way to prevent the hand from grasping it as if great force were to be exerted. It is flat to allow the tips of the fingers merely to touch it, to keep it from turning or rotating upon itself. The brush is narrower at the end or tip since the molar teeth are so much shorter than the others

With such a brush, the teeth would soon be slaughtered if some special way of using it be not devised. *It is on just this point that all persons need to be educated.* The plan cannot be well applied with the old style brush. My theory is the ends of the stiff bristles should be simply laid on the surface of the teeth and without forcing them across, press them down upon the faces of the teeth and force the bristles between them like so many tooth picks, pricking or piercing the particles on or between them, at the same time make the slightest rotary motion, and never, at any time enough to force them out of the spaces between the teeth. When one part is done go around the mouth on all the surfaces. The arrangement and distribution of the bristles is such that the small handle is sufficient to press the bristles home with ease and make it impossible to inflict any injury upon teeth or gums.

To still further make the friction less, the brush should be used immediately after a meal and never before, while the accretions upon the teeth are yet soft. One very light brushing at the proper moment will be the most effective.

While Brush No. 2 will cleanse the palatal and lingual surfaces well, it can be done better with Brush No. 1, which is much smaller. If I were compelled to make use of one only, I would take the smaller, No. 1.

This *new feature* of direct pressure without lateral movement, can only be done with a small brush—short and narrow, and but few and stiff bristles.

Here is where the mischief is done from having to force such a dense body of bristles on and between the teeth. This baby-brush meets the issue.

It is claimed that powders do much injury. I do not see it. Think again! The dry powder is taken upon the moistened brush and as soon as it is in the mouth the saliva floods the brush and the powder drops on the tongue, unless the brush is a very soft one. The mischief is done by the friction of the brush and not by the powder. If we must use powders (and I grant they are very excellent), let us compound them with soap, or something to "hold the powder in solution," as the druggist would say. This would be sure to keep in the brush and do execution without so much friction fron the brush. I know of nothing better than the finest pumice incorporated in fine castile soap. The soap keeps the powder suspended, and, at the same time, prepares the surface of the tooth for the powder to do its work with least abrasion by removing the grease therefrom.

Shall washes be used in conjunction with a brush? I never sold such from my office at any time in my life, nor advised them. If the gums are diseased, no wash, such as is given to patients, will ever cure or harden them. The dentist should first cleanse the mouth and teeth and remove all causes for future trouble, and make one or two applications of concentrated remedies. This will suffice; at least it does with me. I can conceive of no motive in any first-class dentist recommending washes, further than from his ignorance of therapeutics, or a mere love of making sales.

I do not know that I can present the subject more clearly, except by personally demonstrating that the simplicity of their adaption is fully as philosophical as their construction; and the only improvement which I can now see, is to make them yet smaller and the bristles wider apart.

I will further state that the No. 1 is just what we want for our patent rubber and gold plates. It finds the secretions.

Now, gentlemen, I must beg pardon for brushing you up so sharply and consuming so much time over a mere tooth-brush. If I have transcended proper bounds and given you false philosophy, you now have a chance to set aside my teachings.

DISCUSSION.

Dr. Register.—I am pleased with that part of Dr. Bonwill's paper which relates to the curtailing of the brush; I believe that brushes are made too large, but it is folly to assert that they are always the cause of erosion. The reason I asked Dr. Bonwill if his brush would cleanse approximal walls by a wriggling movement, was simply to ascertain the extent of its utility, for it is these walls that we have the greatest difficulty in keeping clean. I do not think a brush can be made to thoroughly cleanse the teeth. The use of compressed air in my practice has forcibly brought to my mind how full of foul matter these interstices are, and I am now advising the use of an atomizer as excellent practice in connection with the brush and floss silk. The evolution of an acid action in an atmosphere of fetid matter takes place principally at night, in the absence of salivary irritation. I do not believe it is a free acid that robs perfect enamel of its lime salts. In using my air apparatus up to 30 to 40 pounds pressure, I am astonished at the unloading of greasy matter from the interstices between the teeth. In pyorrhea alveolaris it removes all foreign matter, so that the tissues have a chance to resume a normal condition. Any atomizer throwing an uninterupted stream will answer. An alkali, such as lime-water, should be used with a special medication when called for.

Dr. James Truman.—Dr. Bonwill's assertion that abrasion is not caused by the tooth brush, is probably true in the sense that the constant use of the bristles over the enamel will not have that effect; but it must be remembered that powder in some form is most always used with the brush, and when used it may cut as sand cuts marble. The objection to powder, and I regard it as a serious one, is the irritation produced upon the gums; this, while scarcely noticeable to the individual, gradually produces absorption at the gingival borders. This in time invites pyorrhea alveolaris and kindred pathological conditions. Greater care is necessary in the use of cleansing agents; charcoal will produce a blueish discoloration of the gums; tooth-picks are terribly destructive to the gum margins and pericementum. Dr. Bonwill's tooth brush No. 1, is a good idea, but neither tooth-powder or pumice should be used with it as a daily practice; the use of them should be mainly confined to rubbing with a piece of soft wood and that directly upon the accretions on the teeth.

Dr. D. Neall.—It seems to me that any process of cleansing the teeth, even by a brush of the most approved kind, which stops short of the thorough cleansing of the approximal surfaces, will be insufficient. The interstices should be kept clean by the daily use of silk thread or even cotton. A simple and small brush, with the handle and back corresponding to the curvature of the teeth, such as I now show you, and which I adopted twenty years ago, should be used. Patients should have impressed upon them that "cleanliness is before godliness"—in care of the teeth and mouth—and all agents and appliances should be simple.

Dr. Tees.—For thirty years I have daily used tooth powder with the brush, in cleansing my own teeth; my children also have used it daily since babyhood, and I fail to find any ill effects from its use. Tooth powders contain but a small quantity of gritty substances, being largely composed of chalk and orris root and but a small proportion of pumice. I do not advise the daily use of soap for cleansing the teeth; on account of its alkalinity, it acts upon the dentine in the same manner as acid acts upon the enamel. I approve of the size of Dr Bonwill's brushes.

Dr. James Truman—The idea that alkaline solutions act upon tooth structure is certainly an erroneous one. I have kept teeth in a strong alkaline solution for over a year, without the slightest effect, either upon the inorganic or basic substance (organic). The enamel was tested by microscopic examination and the latter by decalcification and examination of the resulting tissue. The examination of mouths of persons who have made an habitual use of soap gives the same result. In regard to abrasion ascribed to the tooth brush, I must say that it has a very different origin, which to my mind has not been recognized; it is a prominent cause of caries, but has not been taken into consideration at all. I allude to the change of oral secretions from the neutral to the acid in a state of rest. It is not necessary to enlarge upon this, but I have satisfied myself by careful tests on my own mouth, that a marked acid reaction is the result of a test at this period. The lips hold the oral secretions against the labial surfaces of the teeth, and abrasion results. Antacids are indicated at night more than at any other period in the twenty-four hours, and there is nothing better than *creta prep.* for this purpose.

Dr. E. H. Neall.—In view of the fact that the saliva is frequently in a state of morbid alkalinity, a certain chalk-like decay may thus be accounted for. Dr. Taft says that alkalies will act upon the animal portion of the dentine and remove it, and in cases thus produced, the residue is friable and chalk-like.

Dr. Dixon.—I have seen teeth very much denuded in the mouth of a patient who never used a tooth brush.

Dr. McQuillen.—In my experience, I find the hardest teeth are the most denuded. I saw a patient to-day, whose teeth were badly eroded in places where the brush does not touch.

Dr. Noble.—I have seen the same discoloration of the gums, alluded to by Dr. Truman as being due to the use of charcoal, produced by some systemic cause.—*Dental Office and Laboratory.*

